# Gene panel sequencing in Brazilian patients with retinitis pigmentosa

**DOI:** 10.1186/s40942-017-0087-6

**Published:** 2017-09-11

**Authors:** Kárita Antunes Costa, Mariana Vallim Salles, Chris Whitebirch, John Chiang, Juliana Maria Ferraz Sallum

**Affiliations:** 1 Department of Ophthalmology and Visual Sciences, Federal University of São Paulo (UNIFESP), São Paulo, Brazil; 2 Casey Eye Institute Molecular Diagnostic Laboratory, Oregon Health and Science University (OHSU), Portland, OR USA

**Keywords:** Retinitis pigmentosa, Retinal dystrophies genes, Next generation sequencing, Illumina platform, Molecular diagnosis

## Abstract

**Background:**

Retinal dystrophies constitute a group of diseases characterized by clinical variability and pronounced genetic heterogeneity. Retinitis pigmentosa is the most common subtype of hereditary retinal dystrophy and is characterized by a progressive loss of peripheral field vision (Tunnel Vision), eventual loss of central vision, and progressive night blindness. The characteristics of the fundus changes include bone-spicule formations, attenuated blood vessels, reduced and/or abnormal electroretinograms, changes in structure imaged by optical coherence tomography, and subjective changes in visual function. The different syndromic and nonsyndromic forms of retinal dystrophies can be attributed to mutations in more than 250 genes. Molecular diagnosis for patients with retinitis pigmentosa has been hampered by extreme genetic and clinical heterogeneity between retinitis pigmentosa and other forms of retinal dystrophies. Next generation sequencing (NGS) technologies are among the most promising techniques to identify pathogenic variations in retinal dystrophies.

**Purpose:**

The purpose of this study was to discover the molecular diagnosis for Brazilian patients clinically diagnosed with a retinitis pigmentosa pattern of inheritance by using NGS technologies.

**Materials and methods:**

Sixteen patients with the clinical diagnosis of retinitis pigmentosa were included in the study. Their DNA was sequenced in a panel with 132 genes related to retinal dystrophies using the Illumina^®^ platform. Sequence analysis and variation calling was performed using Soft Genetics^®^, NextGene, and Geneticist Assistant software. The criteria for pathogenicity analysis were established according to the results of prediction programs (Polyphen 2, Mutation taster and MetaCore™) and comparison of pathogenic variations found with databases.

**Results:**

The identified potentially pathogenic variations were all confirmed by Sanger sequencing. There were 89 variations predicted as pathogenic, but only 10 of them supported the conclusion of the molecular diagnosis. Five of the nine patients were autosomal dominant RP (56%), two (22%) were autosomal recessive RP, and two (22%) were X-linked RP. Nine of the 16 patients (56%) had probably positive or positive results.

**Conclusion:**

The Next Generation Sequencing used in this study allowed the molecular diagnosis to be confirmed in 56% of the patients and clarified the inheritance pattern of the patient’s retinal dystrophies.

**Electronic supplementary material:**

The online version of this article (doi:10.1186/s40942-017-0087-6) contains supplementary material, which is available to authorized users.

## Introduction

Inherited Retinal Dystrophies are a group of ocular diseases characterized by slow and progressive vision loss due to the degeneration of specific retinal cells known as photoreceptors. These rare genetic conditions represent the major cause of incurable familial blindness in the Western world leading to visual deficiency, and in some cases, to total blindness [1, 2].

Retinitis pigmentosa (RP, MIM# 268000) is the most common inherited retinal disease. This group of disorders affects 1:3500 individuals in the USA and 1:4000 in Europe [3–8], and is caused by the loss of rod and cone photoreceptor cells. In conjunction with a highly complex phenotype, due to both clinical and genetic heterogeneity, the typical symptoms of RP include: night blindness, decreasing visual fields leading to tunnel vision, and in some instances total blindness. Clinical hallmarks include bone-spicule deposits, attenuated retinal blood vessels, optic disc pallor, visual field loss, and abnormal, diminished, or non-recordable electroretinography responses [5]. Usually the retinal changes begin in the early teenage years and can progress to more severe visual defects at ages between 40 and 50 years, but this evolution can vary widely [9].

Molecular diagnosis of RP is a challenge because this retinal dystrophy can be caused by pathogenic variations in several genes that are also associated with other types of Retinal dystrophies or genes related to syndromic conditions. Retinitis pigmentosa can be expressed in syndromic and non-syndromic forms. In syndromic conditions, RP occurs in addition to abnormalities in non-ocular tissues and organs [4, 9]. The non-syndromic form is more prevalent and accounts for around 65% of all cases [9, 10]. Sporadic cases represent 40–50% of the non-syndromic form [7].

The classical modes of inheritance of RP are autosomal recessive (ARRP), autosomal dominant (ADRP), X-linked (XLRP), and mitochondrial transmission [11–13]. In a Brazilian study, Unonius et al. [14], autosomal recessive RP was the most common type of RP case found, followed by the dominant autosomal form of RP, X-linked RP, and finally isolated cases.

Syndromic and non-syndromic forms of retinal dystrophies together can be attributable to mutations in more than 250 genes. RP can be caused by mutations in upwards of 60 genes, in which 22 genes are related to the dominant form, 36 related to the recessive form, and two genes related to the X-linked form [1, 15, 16].

In this group of heterogenic diseases, different retinal dystrophies may be caused by mutations in the same gene, and one phenotype can be caused by different pathogenic variations in more than one gene [4, 17]. Genes related with syndromic forms of retinal dystrophies may also be related to RP in a non-syndromic form [18].

Molecular diagnosis methods, like Sanger sequencing and Microarray have a low rate of pathogenic variation detection. Sanger sequencing is still the gold standard for DNA sequencing with clinical application. However, there are limitations such as the size of the DNA to be sequenced and the ability to detect multiple variations from one individual. Microarray also has limitations because it only detects known mutations [5].

Genetic and pathogenic variation screening by next-generation sequencing (NGS) is becoming the new gold standard for the genetic diagnosis of retinal dystrophies due the large number of genes involved [19].

The identification of a causative pathogenic variation is important to understand of the genetic basis of the disease. It enables more precise genetic counseling, family planning, and future gene-targeted treatments [20].

The aim of this study was to identify pathogenic variations in the 132 genes known to be associated with retinal dystrophy in 16 Brazilian patients likely to have the autosomal dominant form of RP using next generation sequencing (MiSeq platform by Illumina^®^) technology. The combination of clinical and molecular information gathered using NGS is the most powerful approach to refine the complex diagnosis of RP, and will be critical in guiding the development of gene specific treatments for this, and other, ocular conditions.

## Materials and methods

### Patients

Sixteen Brazilian patients with the clinical diagnosis of non-syndromic retinitis pigmentosa that could likely have the autosomal dominant subset pattern of inheritance were analyzed. The clinical diagnosis of RP was established by ophthalmological examination based on the presence of night blindness, progressive peripheral vision loss decreasing visual acuity, and subsidiary exams as needed. All patients were recruited from the Department of Ophthalmology, Federal of São Paulo University/Brazil. Pedigrees were established based on patient interviews.

### Genomic DNA extraction and sample preparation

Genomic DNA of patients was extracted from peripheral blood using standard protocols (Blood DNA midi kit, Qiagen^®^, USA) following the manufacturer’s instructions.

Primers for all coding and noncoding exons, including 50 bp of flanking 5′ and 3′ intronic sequence, were designed using the Primer 3 program (available in the public domain at http://bioinfo.ut.ee/primer3-0.4.0/). For amplification, we applied a PCR protocol using 100 ng of genomic DNA in a total volume of 25 µl. Thermal cycling was performed with the touchdown conditions.

Illumina libraries (Illumina^®^, San Diego, CA, USA) were generated according to the manufacture’s standard protocol for genomic DNA library preparation.

Adaptors were ligated at the 3′ and 5′ end of each DNA fragment for sequencing. There are three important regions present in these adapters: (1) region complementary to oligonucleotides of the flow cell, (2) complementary region to sequencing primers and, (3) complementary region to the primers of the bar codes. These bar codes were sequenced and read along with each sequence in order to identify each patient at the end of the sequencing. The preparation of the library and coupling of the bar codes followed the Illumina^®^ protocol.

Equal molar ratios of all samples were pooled and checked by real time quantitative qPCR (ViiA™ 7 Real-Time PCR System, Thermo Fisher Scientific). This dilution procedure is important before sequencing on a MiSeq platform in order to ensure correct DNA concentration.

After the PCR procedures, all samples were combined together to be applied to the MiSeq sequencing protocols (Illumina^®^, San Diego, CA, USA).

### Panel sequencing

The panel with 132 retinal dystrophy genes was developed at the Casey Eye Institute (CEI) Molecular Diagnostic Laboratory. The 132 gene panel was comprised of coding exons and splicing sites of 132 known retinal disease genes. These 132 genes are related to all non-syndromic and a few syndromic forms of RP (Table 1).

**Table 1:** 132 Genes included in the panel to retinal dystrophies (RD/132 panel, year 2014)

RP panel with 132 genes	
ABCA4, ABHD12, ADAM9, AIPL1, ALMSI, ASCC3L1/SNRNP200, BBS1, BBS2, ARL6/BBS3, BBS4, BBS5, MKKS/BBS6, BBS7, BBS9, BBS10, TRIM32/BBS11, BBS12, BBS13/mksl, WDPCP/BBS15, SDCCAG8/BBS16, LZTFL1/BBS17, BEST1, C1QTNF5, C2orf71, C8orf37, CA4, CABP4, CACNA2D4, CACNA1F, CDH23, CDHR1, CEP290/BBS14, CERKL, CHM, CLRN1, CNGA1, CNGA3, CNGB1, CNGB3, CNNM4, CRB1, DFNB31, DHDDS, ELOV4, EYS, FAM161A, FSCN2, GNAT1, GNAT2, GPR98, GPR179, GRM6, GUCA1A, GUCA1B, GUCY2D, HARS, IDH3B, INPP5E, IMPDH1, IMPG2, IQCB1, KCNJ13, KLHL7, LCA5, LRAT, LRIT3, MAK, MERTK, MYO7A, NMNAT1, NR2E3, NRL, NYX, OFD1, OPA1, OPA3, OTX2, PCDH15, PDE6A, PDE6B, PDE6C,PDE6H, PDE6G, PDZD7, PITPNM3, PRCD, PROMl, PRPF3, PRPF6, PRPF8, PRPF31, RAX2, RBP3, RD3, RDH5, RDH12, RDS/PRPH2, RGR, RHO, RIMSI, RLBP1, ROM1, RP1, RP1L1, RP2, RP9, RPE65, RPGR, RPGRIP1, RS1, SAG, SEMA4A, SLC24A1, SPATA7, TOPORS, TRPM1, TTC8/BBS8, TULP1, USH1C, USH1G, USH1J-CIB2, UNC119, USH2A, ZNF513, NPHP1, CYP4V2, PEX7ASTN2, PHYH, MFRP, CYGB, TFPT	

Capture target libraries from 16 Brazilian probands were sequenced using next generation sequencing (MiSeq platform by Illumina^®^) and V2 reagent kits (Illumina^®^, San Diego, California, USA) according to the standard operating protocol to obtain sizes of fragments between 250 and 300 pb paired-end reads. All protocol details of panel design, library preparation, capture sequencing, and variant analysis were developed by the CEI team along with the scientific advice of Illumina^®^.

### Bioinformatics analysis

Because of the large volume of information obtained by next generation sequencing, dedicated bioinformatics resources are required to fully utilize the results.

First, sequences obtained were compared to human references genome using NextGene software for next generation sequencing analysis (SoftGenetics^®^, State College, PA, USA).

Then, all the differences compared to the reference sequence were analyzed by prediction programs (Polyphen 2, Mutation Taster, MetaCore™).

Finally, the databases of the National Center for Biotechnology Information (NCBI), the Online Mendelian Inheritance in Man (OMIM) [21], the Human Gene Mutation Database (HGMD) [22], Ensembl, 1000 Genome Browsers, and ExAC Browser were used to analyze the variants.

Prediction programs were used to calculate scores, based on different algorithms, to classify the variations identified as pathogenic or benign. The possible pathogenicity of missense variants was predicted using Polyphen 2 program (http://genetics.bwh.harvard.edu/pph2/) and MetaCore™ (http://lsresearch.thomsonreuters.com/pages/solutions/1/metacore). Mutation tester software was used to find intronic and synonymous variations (http://www.mutationtaster.org).

All identified variants were classified into three categories: pathogenic, benign, and unknown. This bioinformatics analysis was also based on and compared with the Casey Eye Institute [(CEI) Oregon Health and Science University] database protocols described in the CEI manual of standard operating procedures, and by using some information from American College of Medical Genetics and Genomics (ACMG) [23].

Pathogenic variations already reported in the literature were classified as positive results. For pathogenic variations that had not been reported in literature, the result was considered probably positive (Table 2).

**Table 2:** Conclusive results in 9 (56%) of 16 patients

Patient	Age	Gender	Gene/disease causing variation	Gene/non pathogenic variation	Results
5	64	M	CRB1/c.1436 T > C Leu479Pro Homozygous+	CNGA1/c.1315G > A Val439Met (heterozygous) FSCN2/c.229G > A Val77Met (heterozygous) PHYH/c.734G/A Arg245Gln (homozygous)	Probably positive (ARRP)
9	27	F	ROM1/c.671C > T Pro224Leu Heterozygous+	ABCA4/c.3759G > A p. Thr1253Thr (heterozygous) CEP290/c.1298A > G p. Asp433Gly (heterozygous) GRM6/c.1791C > T Ille597Ille (heterozygous) OPA3/c.135G > A p. Pro45Pro (heterozygous) OTX2/c.126C > A p. Thr42Thr (heterozygous) PITPNM3/c.1671C > T p. Tyr557Tyr (heterozygous) PRPF8/c.708G > A p. Ser236Ser (heterozygous)	Probably positive (ADRP)
12	35	F	PDE6B/c.3G > T p. Met1Ile, and c.313G > A p. Glu105Lys Heterozygous+	ABCA4 c.1029T > C p. Asn343Asn (heterozygous) CDHR1 c.1849G > A p. Ala617Thr (heterozygous) USH2A c.12823T > A p. Ser4275Thr (heterozygous) ZNF513 c.96G > C p. Leu32Leu (heterozygous)	Probably positive (ARRP)
15	53	M	SNRNP200/c.2359G > A p. Ala787Thr Heterozygous+	CNGA3 c.943G > A p. Asp315Asn (heterozygous) GRM5 c.1732C > T p. Arg578Cys (heterozygous) MERTK c.61 + 3G > C (heterozygous) PCDH15 c.2596G > A p. Val866Met (heterozygous) SEMA4A c.405T > C p. Asn135Asn (heterozygous)	Probably positive (ADRP)
16	39	M	PRPF31/c.906_907insGCCAAGTGCACACTGGCAGCC Heterozygous+	BBS12 c.116T > C p. Ile39Thr (heterozygous) CDH23 c.7722C > T p. Tyr2574Tyr (heterozygous) RP1L1 c.4620G > C p. Glu1540Asp (heterozygous) USH2A c.2276G > T p. Cys759Phe (heterozygous)	Probably positive (ADRP)
6	39	M	RPGR/c.905G > C p. Cys302Ser Hemizygous+	CRB1/c.614T > C p. Ile205Thr (heterozygous) CYP4V2/c.40C > G p. Leu14Val (heterozygous) PRPF8/c.4707G > A p. Leu1569Leu (homozygous) RD3/c.560C > A p. Pro187His (heterozygous) RPGRIP1/c.3402_3404delGTC (heterozygous)	Positive (XLRP)
8	40	M	RPGR/c.1243_1244delAG Hemizygous+	BBS12/c.355G > A p. Gly119Ser (heterozygous) CDH23/c.6852G > C p. Leu2284Leu (heterozygous) USH1C/c.566G > A p. Arg189Gln (heterozygous)	Positive (XLRP)
10	37	F	RHO/c.568G > A p. Asp190Asn Heterozygous+	CNGB1/c.2681G > A p. Arg894His (heterozygous) CRB1/c.614T > C p. Ile205Thr (heterozygous) GPR179/c.4597G > A p. Glu1533Lys (heterozygous) GUCY2D/c.2765A > G p. Tyr922Cys (heterozygous)	Positive (ADRP)
14	54	M	NR2E3 c.166G > A p. Gly56Arg Heterozygous+	ALMS1 c.5294A > G p. Asn1765Ser (heterozygous) CACNA1F c.5050G > T p.Gly1684Trp (hemizygous) CDH23 c.6713-8G > A (heterozygous) CDH23 c.10026C > T p. Asp3342Asp (heterozygous) CEP290 c.1716A > G p. Leu572Leu (heterozygous) CEP290 c.1558T > C p. Phe520Leu (heterozygous) CNGB1 c.165C > T p. Pro55Pro (heterozygous) FAM161A c.1851 + 22G > A (heterozygous) FSCN2 c.576C > T p. Arg192Arg (heterozygous) LCA5 c.1323C > T p. Tyr441Tyr (heterozgous) RBP3 c.3546C > T p. His1182His (heterozygous) USH2A c.14226G > A p. Thr4742Thr (heterozygous)	Positive (ADRP)

Molecular features of 9 retinitis pigmentosa patients with probably positive and positive results

*F* female, *M* male, *ARRP* autosomal recessive retinitis pigmentosa, *ADRP* autosomal dominant retinitis pigmentosa, *XLRP* X-linked retinitis pigmentosa

### Validation

All candidate variations classified initially as pathogenic, probably pathogenic, or unknown were confirmed by Sanger sequencing. For mutation confirmation and gaps covered by Sanger sequencing, specific primers were designed for polymerase chain reaction (PCR) amplification using Primer 3 (v.0.4.0) software (http://bioinfo.ut.ee/primer3-0.4.0/).

## Results

The MiSeq output from kit V2 generated 7.5–8.5 Gb with 250–300 pb read length. The results of NGS screening in our cohort of 16 patients are summarized in Tables 2 and 3. In total, 9.707 variations were identified including benign, pathogenic, and unknown variations (Table 4). Automated variant detection for all 132 genes resulted in an average of 607 variations per sample between benign and candidate variations. Only candidate variations (probably pathogenic and unknown) were analyzed for pathogenicity.

**Table 3:** Pathogenic variations non-causing of retinitis pigmentosa

Patient	Age	Gender	Gene/disease causing variation	Gene/pathogenic non disease causing variation	Results
1	43	F	None	BBS9/c.666T > A p. Thr222Thr (heterozygous) BBS9/c.2214C > T p. Ile738Ile (heterozygous) PCDH15/c.608C > T p. Pro203Leu (heterozygous) SAGc.473C > A p. Thr158Lys (heterozygous)	Inconclusive
2	47	F	None	CNGA3/C.942C > A p. Thr314Thr/Lys (heterozygous) IQCB1/c.1393T > C p. Tyr465His (heterozygous) MFRP/c.771C > T p. Arg257Arg (heterozygous) MKS1/c.496C > T p. Arg166Cys (heterozygous) USH2A/c. 13355T > C p. Leu4452Ser (heterozygous)	Inconclusive
3	73	M	None	CRB1/c.3210 C > A p. Ser1070Arg (heterozygous) TRPM1/c. 1846-8C > G (heterozygous) TRPM1/c. 1453G > C p. Glu485Gln (heterozygous)	Inconclusive
4	17	F	None	GPR98/c.7569A > G p. Thr523Thr (heterozygous) GRM6/c.1706C > T p. Thr569Met (heterozygous) LCA5/c.609A > G p. Leu203Leu (heterozygous) PCDH15/c.2578C > T p. Arg860Trp (heterozygous) SAG/c.271C > T p. Arg91Trp (heterozygous) SDCCAG8/c.968G > A p. Arg323Lys (heterozygous) SLC24A1/c.2002C > T p. Arg668Cys (heterozygous)	Inconclusive
7	59	F	None	GUCY2D/c.2179G > A p. Gly727Ser (heterozygous) MKKS/c.760G > T p. Asp254Tyr (heterozygous) PDE6B/c. 1653C > T p. Tyr551Tyr (heterozygous) PDE6C/c.986A > C p. Glu329Ala (heterozygous) RBP3/c.2556C > T p. Ala852Ala (heterozygous) RIMS1/c.3959C > A p. Ser1320Tyr (heterozygous)	Inconclusive
11	20	F	None	CDH23 c.367G > A p. Gly123Arg (heterozygous) CDH23 c.7132G > A p. Gly2378Arg (heterozygous) CDHR1 c.155C > T p. Ser52Phe (heterozygous) CERKL c.589G > T p. Ala197Ser (heterozygous) FAM161A c.1133T > G p. Leu378Arg (heterozygous) RPGRIP1 c.256C > T p. Arg86Trp (heterozygous) USH2A c.5039A > G p. Lys1680Arg (heterozygous)	Inconclusive
13	39	M	None	N/A	Negative

*N/A* not applicable, *F* female, *M* male

**Table 4:** Total of 9.707 variations found (benign, pathogenic and unknown)

Patient	Coverage of gaps (%)	Varistions found	Variations confirmed by Sanger sequencing	Intronic variations	Missense variations	Deletions	Insertions	Synonymous variations
1	73.0	557	7	2	2	0	0	3
2	93.0	573	13	6	5	0	0	2
3	94.4	634	8	3	4	0	0	1
4	96.0	632	19	4	12	0	0	3
5	91.0	391	10	1	8	0	0	1
6	95.0	623	13	6	6	1	0	0
7	86.0	638	17	4	7	2	0	4
8	95.0	697	12	2	6	2	0	2
9	93.0	622	15	4	4	0	0	7
10	85.0	660	9	3	6	0	0	0
11	95.0	590	9	0	9	0	0	0
12	95.0	554	9	1	5	0	0	3
13	95.0	691	2	0	2	0	0	0
14	92.0	685	23	7	6	1	1	8
15	94.0	564	8	3	4	0	0	1
16	93.0	596	11	1	6	0	1	4
Total = 16	Average coverage of Gaps = 92	9.707	186	47	92	6	2	39

Classification of the variations found: total of 186 variations candidates to be causative to retinitis pigmentosa and confirmed by Sanger sequencing, 47 intronic variations, 92 missense variations, 6 deletions and 2 insertions, coverage gaps with an average of 92% achieved by Sanger sequencing after next generation sequencing and 39 synonymous variants identified in this study

All exons containing any base with less than 30X of coverage by NGS, called gaps, were completed by new specific PCR amplification and Sanger sequencing of the particular coding sequences (CDS). Gaps are DNA regions not covered by next generation sequencing platforms. This limitation was solved using a second sequencing method called Sanger sequencing. This second sequencing method is usually used to validate pathogenic variations and cover the gaps. An average of 92% of gap coverage was achieved with Sanger sequencing (Table 4).

From the 9.707 variations found, 186 were candidates to be the causative pathogenic variations. Those were confirmed by Sanger sequencing and analyzed by prediction programs (Polyphen2, Mutation Taster and MetaCore™) and selected databases (NCBI, 1000 genomes, HGMD). In total, 92 missense variations, 47 splice-site alterations, 6 deletions, and 2 insertions were analyzed (Table 4, and more details shown in Additional file 1: Table).

The clinical diagnosis was reviewed with the genetic diagnosis, and if necessary, a subsequent clinical reassessment, to confirm if the genetic diagnosis found using NGS was compatible with the clinical characteristics.

A total of 10 disease causing variants were identified in this cohort of 16 patients (Table 2), allowing the molecular diagnosis of nine patients (56%). After obtaining the DNA results, the pattern of inheritance could be more precisely defined. Those 16 patients could have had an autosomal dominant pattern of inheritance, but it was impossible to exclude the autosomal recessive or X-linked patterns. Five of the nine patients were autosomal dominant RP (56%), two (22%) were autosomal recessive RP and two (22%) were X-linked RP.

The diagnostic yield of 56% was attributed to Sanger validation, satisfactory coverage, high quality data, sensitivity, and specificity of the method.

Five patients were diagnosed molecularly, however the term “probably positive” remains since their results were pathogenic variations that were not previously reported in the literature, nor in mutation databases.

Patient 12 was considered “Probably positive” due to the fact just one variation predicted as likely pathogenic has been found. The PDE6B gene is related to ARRP (Table 2).

Patients 5, 9, 15 and 16 also had the probably positive result. Patient 5 had one homozygous variation in the CRB1 gene found. CRB1 is a gene related to ARRP and the variation found wasn’t previously reported (Table 2).

Patients 9, 15, and 16 had one pathogenic variation found in ROM1, SNRNP200, and PRPF31 gene respectively. All of these three genes are associated with autosomal dominant RP. Although each patient has one mutation in a dominant gene, these variations were not found in the literature and thus the term “probably positive” remains in the report (Table 2).

Fifteen patients had heterozygous variations predicted as pathogenic in one or more genes associated with autosomal recessive RP or another retinal dystrophy (Additional file 1: Table). All inconclusive and negative patients (44%) were heterozygous carriers for variations predicted as pathogenic in one or more recessive retinal genes, but this could not confirm the molecular diagnosis.

Patient 13 had a negative result because all of his identified variations were predicted as benign. Molecular diagnosis for this patient remains unclear (Table 3).

Our results provide relevant information of variants found in a cohort of RP Brazilian patients and increases our knowledge of molecular findings related to RP.

Almost half of RP cases are isolated cases in which the inheritance pattern cannot be reliably determined.

Clinical analysis, in combination with pedigree information and molecular data, enabled the confirmation of RP diagnosis in all these patients. However, a homozygous pathogenic variation c.1436T < C Leu479Pro in CRB1 gene, a heterozygous pathogenic variation c.671C > T p. Pro224Leu in the ROM1 gene, two heterozygous pathogenic variations, c.3G > T p. Met1Ile and c.313 G > A p. Glu105Lys in PDE6B gene, a heterozygous pathogenic variation c.2359 G > A p. Ala787Thr identified in SNRNP200 gene and heterozygous pathogenic variations c.906_907insGCCAAGTGCACACTGGCAGCC in PRPF31 gene were identified, but it was prudent to conclude that all of the patient results were probably positive even though the variations predicted as pathogenic were not found in the literature (Table 2).

## Discussion

Molecular diagnosis of retinal diseases is complex due the large number of related genes and the overlapping of the clinical characteristics. However, molecular diagnosis is essential for accurate clinical diagnosis, more precise genetic counselling, and treatment development [20].

Variation was the term used in this work for any nucleotides change in the sequence of DNA of all patients analyzed. Mutation is a change in the nucleotide sequence, and polymorphism is defined as a variant with a frequency above 1%. The terms “mutation” and “polymorphism” are used widely, however this can lead to confusion due to incorrect assumptions of pathogenic and benign effects, respectively. In their standard guidelines, Sue Richards and collaborators [23] recommend the use of specific standard terminology according with each laboratory practice. These researchers also recommend that each research center should apply their own professional judgment to specific circumstances adopted.

Nine patients had a positive or probably positive result (Table 2). For patients 5, 9, 12, 15 and 16, the term “probably positive” was kept since variations were not reported before. These results may suggest that these pathogenic variations are novel (Table 2).

For patient 9, the variation predicted as pathogenic allowed the molecular diagnosis conclusion to be probably positive for the autosomal dominant form of RP. This patient, initially diagnosed with autosomal dominant RP, displayed clinical features similar to cone rod dystrophy. The molecular information established by the results of NGS helped in refining the clinical diagnosis of this patient and confirmed the pattern of inheritance as ADRP.

Patients 6 and 8 had the positive molecular diagnosis of the RPGR gene. This gene is related to X-linked RP. The pathogenic variation c.905G > C p. Cys302Ser in the RPGR gene identified in patient 6 supported a positive result. This pathogenic variation is already described in the literature [24]. This molecular information combined with clinical information confirms the genetic diagnosis. A pathogenic variation c.1243_1244delAG found in RPGR gene in patient 8, as well as pathogenic variation c.568G > A p. Asp190Asn in the RHO gene (Rhodopsin gene) in patient 10 and pathogenic variations, c.166 G > A p. Gly56Arg in NR2E3 gene in patient 14, allowed the positivity of the test and conclusion of the genetic diagnosis for all these patients. All of these molecular data are already reported in the literature [25–27].

Results from the present study demonstrate the importance to group together molecular and clinical information in order to conclude a molecular and clinical diagnosis. Clinical and genetic factors, in combination, allowed conclusive results in 9 out of 16 patients (56%) in this study.

Seven patients (44%) remained unsolved due to a variety of reasons. For example, the panel used in these patients was limited to 132 genes (Tables 3, 4). Now, the panel currently has more than 250 genes [15], which may hold the answers for some of these patients.

Differences in data quality, insufficiently covered sequences, the presence of deep intronic mutations causing aberrant splicing, mutations in regulatory regions where not targeted by the 132 genes panel, pathogenic variation in a gene not currently associated with RP, epigenetic mechanisms, syndromic genes, uncertain clinical diagnoses, and uncharacterized regions can also be reasons for unsolved cases [1, 5, 19].

Eleven of the 16 patients (69%) demonstrated variations predicted to be pathogenic in genes associated with syndromic conditions, such as USH2A, BBS9, PCDH15, GPR98, CEP290, BBS12, USH1C and MKKS (Tables 2, 3). These genes are related to recessive diseases. Aside from that, only one variation predicted as pathogenic was found for each.

Pathogenic variations in the BBS1 gene, previously known to cause Bardet Biedl syndrome, was recently identified in RP patients in a non-syndromic form [18]. Also, pathogenic variations in BBS6/MKKS were related to non-syndromic RP [28]. Similar cases happened in other studies developed by Wang et al. [17] in which mutations in the CLN3 gene in patients without syndromic characteristics of different types of retinal dystrophies, including RP, suggest that CLN3 is also a non-syndromic retinal disease gene. This data indicates that mutations in CLN3 can cause non-syndromic retinal degeneration, which implies a more favorable prognosis for patients carrying these mutations in CLN3.

The USH2A gene was one of the genes with more variations predicted as pathogenic in our patient cohort. Although this gene is associated with the non-syndromic form on RP, patients with variations classified as pathogenic for this gene showed only one variation. This gene is associated with the recessive form of RP and also with Usher syndrome.

Digenic events, simultaneous presence of heterozygous mutations in two autosomal dominant genes or “double hit” with mutations in two or more autosomal recessive RP genes, are other genetic mechanisms in these heterogeneous diseases [29–31].

Recently, a rare combination of mutations in ABCA4 and GRM6, genes whose mutations are associated with more than one form of retinal dystrophies, was reported in a patient with atypical Stargardt disease [32]. A highly variable phenotype and progression of some retinal dystrophies, like Stargardt disease, have been documented, and mutations in the ABCA4 gene have also been implicated in cone-rod dystrophy and retinitis pigmentosa. The clinical and genetic overlap between RP and other retinal diseases is extremely complex [11, 19, 24, 33, 51].

The importance to correlate the pathogenic variations with genesis or severity of genetic diseases makes the NGS technique a great tool that allows the identification of variations in many genes at the same time. There are three NGS strategies: Whole Exome Sequencing (WES) that involves the capture of all exons which leads to identification of several RP genes and novel mutations with a lower sensitivity compared to panels [34–38], a Whole Genome Sequencing (WGS) technique used to cover nearly all the human genome with the main limitation being the cost [39], and a third NGS strategy called “Targeted Capture” which was used in this study. This strategy limits testing to exons of known disease-causing genes [40]. Despite the disadvantages that no new genes could be identified, the advantages are that the analysis “space” is much smaller, more is known, a priori, about each gene which makes the strategy appropriate for screening for RP [41, 42]. One additional advantage for panel testing is that modifiers, digenic mutations, and multiallelic interactions can also be identified through panel testing [19].

Variations in some complicated genes wasn’t accessible due to highly repetitive sequence of single nucleotide or blocks. This prevented the range of 100% coverage of all coding regions of the genes in this 132 gene panel (Table 4). The hot spot of ORF15 in RPGR gene was one of these situations. The mutational hot spot exon of RPGR, ORF15, was not accessible by our sequencing approaches in all cases due to its highly repetitive sequence. This problem happened at the time the test was performed, but has since been recently solved. This problem also occurred in other studies based on NGS screening [1, 4, 43].

Many novel variations are listed in private databases and are not yet in the public domain. This is another problem faced by researchers hoping to establish the correct molecular diagnosis [44].

Although Sanger sequencing is the gold standard for genetic diagnosis, with a few exceptions, there are no ophthalmologic characteristics specifically associated with genetic subtypes of RP, precluding the prioritizations of genes to be analyzed by this technology [45]. NGS is at least 1000 times faster than conventional sequencing, and much less expensive per nucleotide sequence [4].

Combining results from conventional Sanger sequencing and Targeted Capture NGS, using rough estimates, it is possible to detect the underlying pathogenic variations in a good percentage of cases. In recent studies, detection of pathogenic mutations searched 20–30% for the autosomal recessive RP cases, 60–70% for autosomal dominant cases, 80–85% for X linked cases, and more than 85% for Usher and BBS cases [4, 46].

### Synonymous variations

All synonymous variations were predicted in this study. Synonymous variations are classified for being of little importance once that they change the nucleotide, but do not change the amino acid. However, recent studies have questioned the pathogenicity of these variations and their action both at the level of transcription and protein folding. Although there are studies to understand if synonymous variants are diseases causing or not for certain genetic diseases [47], in this work, 39 synonymous variations were identified, but were not clinically interpreted (Table 4).

### Future prospects for studies in the Brazilian population

After a great number of patients are analyzed and correlated genotypic and phenotypically, the association between certain variations, not only with the genesis of the disease but also to the severity, may be established. The databases can be improved with the inclusion of more results and this will improve the detection rate of the pathogenic variations.

Compared with the efficiency of results from these important studies, this study also supports the efficiency of the NGS method as the screening method of choice for complex and genetically heterogeneous subtypes of retinal dystrophies, such as RP, in a genetically unknown population.

Wang et al. [48] concluded that the mutation spectrum in the Chinese population is distinct compared to that in the European population which makes NGS a more efficient tool in terms of numbers of sequenced genes.

Weisschuh et al. [1], in a cohort of 89 unrelated cases were able to identify coding mutations in 52 cases and non-coding mutations in two cases, corresponding to 5% of previously unsolved cases. This confirms the need for analysis of regions outside of the coding exons. Their studies also confirmed the diagnostic value of NGS platforms in the identification of pathogenic variations in a heterogeneous disease like retinal dystrophy.

A recent study using a panel of 66 genes reported a diagnostic yield of 82% [49]. In another study, a panel of 55 genes reported a diagnostic yield of 70%. However, the number of genes present on the panels for retinal dystrophies recently increased to approximately 250 genes [15]. This figure reports the mean increase of the resolution of genetic tests when the genes included in the panel increases [17, 41, 43, 50].

The efficiency of NGS to identify pathogenic variations was confirmed in the studies cited above, corroborating with the present study which was able to establish the genotyping in 56% of the patients analyzed.

Molecular diagnosis does not depend solely on pathogenic variation identification, but also on clinical information. Pedigree is extremely important to understand the allele segregation. In almost half of RP cases, the inheritance pattern cannot be reliably determined due to limited pedigree size [12]. More sequencing, and consequently more information about specific genes and variations associated with type and subtype of retinal dystrophies, can perform a comprehensive molecular diagnosis to include both known RP genes and other retina disease genes.

There is no effective cure for retinal dystrophies, however, ongoing clinical trials applying gene-replacement therapy approaches for several forms of retinal dystrophies have raised new hopes. Since these approaches require the identification of the causative pathogenic variations, the molecular diagnosis is an essential prerequisite [1].

Genetic testing, and consequently the molecular diagnosis, allows for more precise genetic counselling due to the fact that it helps to better define the pattern of inheritance in the family. Molecular diagnosis is important to establish a complete and efficient characterization of the patients, allowing each patient to receive a more specific prognosis, and this goes for their families as well. Patients with a conclusive molecular diagnosis may benefit from appropriate genetic counseling, and can be included in studies for therapies for specific genes or specific pathogenic variations.

## Conclusion

This study demonstrates that next generation sequencing offers an effective method for the molecular diagnosis of Retinitis Pigmentosa. Nine (56%) Brazilian patients had their molecular diagnosis established. These results highlight the importance of a molecular diagnosis as an integral part of the clinical diagnostic process. It provides a more accurate clinical diagnosis and allows for efficient genetic counseling, family planning, and future gene-targeted treatment.

## Additional file

**Additional file 1.**Variation identified in all 16 patients. All variations were predicted as pathogenic or benign, except classified as benign.
