# Supplementary material for: Gene panel sequencing in Brazilian patients with retinitis pigmentosa
Source: Int J Retina Vitreous. 2017 Sep 11;3:33. doi: 10.1186/s40942-017-0087-6 (PMC5592712; doi:10.1186/s40942-017-0087-6)
Supplement: Supplementary file 1 — Additional file 1.Variation identified in all 16 patients. All variations were predicted as pathogenic or benign, except classified as benign. [file 40942_2017_87_MOESM1_ESM.docx]

**Supplementary table**: Variation identified in all 16 patients. All variations were predicted as pathogenic or benign, except classified as benign.

**Note Supplementary table:** Rec. (Recessive gene for highlighted condition), Dom (dominant gene for highlighted condition), CSNB (Congenital Stationary Night Blindness), CRD (cone Rod Dystrophy), RP (Retinitis Pigmentosa), Pat (pathogenic), Pol (Polymorphism) LCA (Leber Congenital Amaurosis). Grey highlighted: Variations classified pathogenic. Bold and underlined variations: pathogenic variations that made possible the molecular diagnostics conclusion.
